# Effects of Mechanical Damage for Different Type of Threshing Patterns on Wheat Storage Quality Traits

**DOI:** 10.3390/foods14091577

**Published:** 2025-04-30

**Authors:** Ling Li, Yinian Li, Yulun Chen, Qishuo Ding, Ruiyin He, Yingying Liu

**Affiliations:** 1 College of Engineering, Nanjing Agricultural University, Nanjing 210031, China; FuLImu2018@163.com (L.L.); chenyulun@njau.edu.cn (Y.C.); qsding@njau.edu.cn (Q.D.); ryhe@njau.edu.cn (R.H.); 2 College of Artificial Intelligence, Nanjing Agricultural University, Nanjing 210031, China

**Keywords:** wheat, tangential flow threshing pattern (TFTP), axial flow threshing pattern (AFTP), threshing damage, grain damage, mold colonies, storage quality

## Abstract

Mechanical damage to wheat grains is inevitable during harvester threshing; damaged wheat grains are vulnerable to mold colonies. Selecting optimal threshing pattern is an important approach to reduce wheat storage quality loss for threshing damage. Wheat harvested by tangential flow threshing patterns (TFTP) and axial flow threshing patterns (AFTP) were used as test samples and wheat harvested manually were used as control samples. Moisture contents of all wheat grains were adjusted to 18% (wet basis) before storage, then wheat grains were stored in an artificial climate box at temperature of 30 °C and humidity of 80% RH. The mold colonies and fatty acids value of wheat harvested by TFTP increased faster than those of wheat harvested by AFTP, the dry basis 1000 grain weight, seed vigor and germinating traits of wheat harvested by TFTP decreased faster than those by AFTP during storage. After storage, there were significant differences between TFTP and AFTP in the mold colonies, seed vigor, dry basis 1000 grain weight (*p* < 0.05). Germination potential and rate were significantly negative correlation with mold colonies. Aberrations of seedling and mold colonies in seedling root had significantly positive correlation with the mold colonies. The storage quality traits of wheat harvested by AFTP were better than those of wheat harvested by TFTP. AFTP should be preferred for wheat harvested mechanically in terms of the storage quality of wheat.

## 1. Introduction

Wheat grains occur mildew under severe storage conditions, mildew of wheat grains is mainly caused by mold, which is an important factor in deteriorating wheat quality [1]. Mold colonies of wheat grains are used to decide the mildew levels [2], the mold colonies are less than 10^3^ cfu·g^−1^, wheat grains are in safe storage; the mold colonies reach 10^3^ cfu·g^−1^, wheat grains mildew mildly; the mold colonies reach 10^4^ cfu·g^−1^, wheat grains mildew moderately; the mold colonies reach 10^5^ cfu·g^−1^, wheat grains mildew severely and the storage quality of mildewed wheat reduces [3,4,5,6]. Factors that affect storage quality of wheat include the moisture content of grains, environmental temperature and humidity. At present, mold activities of wheat grains under different storage conditions [7] and mold activities of different variety wheat grains [8] are researched after threshing, and the effects of mold activities on the storage quality of wheat grains also are analyzed [5]. In order to elucidate the influence mechanism of fungal action on wheat grains quality, the research on the diversity and distribution of fungal in storage wheat grains are conducted [6,9,10,11]. The research results reveal significant differences in the fungal diversity and communities at different stages of wheat processing, and a more complex fungal co-occurrence network in stored wheat than in harvested wheat [6]. Different fungi tend to consume different wheat components, and the interaction between environmental and biological factors eventually leads to the deterioration of wheat quality [12]. In order to compromise the storage quality of wheat grains, the reducing competition between fungal communities could prevent mildew infestations and mycotoxins contamination of wheat grains [13]. Environmental factors can affect the wheat mycobiome assembly, and wheat mycobiome mediates the interaction between environmental factors and mycotoxin contamination [14]. Controlling moisture of wheat grains during the storage could decrease the nutrient loss and cell damage [15]. Low storage temperatures and humidity result in low levels of Fusarium mycotoxins.

Crop grains are harvested by combine harvester at present, but machinery harvester inevitably damage crop grains during threshing process [16,17]. In order to reduce the mechanical damage, designing new threshing machines and optimizing operating parameters of harvester are performed [18,19]. On the other hand, effects of threshing damage on quality properties of crop grains are also investigated [20]. These effects include seed vigor, germination rate, processing quality and storage quality [21,22]. Mildew easily infects the damaged crop grains, quality of grains reduces quickly and storage property of damaged crop grains deteriorates quickly [23].

Two main mechanically threshing patterns, tangential flow threshing (TFT) and axial flow threshing (AFT), are used in harvesting wheat grains at present [24]. Wheat grains are damaged and broken during the mechanical threshing process of the wheat grains [24,25,26,27]. Mechanical damage to wheat grains is inevitable, and damaged wheat grains are more vulnerable to mold colonies during the storage process [28], which accelerates the deterioration of wheat quality. This study researches the threshing damage effects of different types of threshing patterns to wheat grains from the storage quality traits.

In this study, wheat grains harvested by two main threshing patterns, TFT and AFT, are used as test samples and manual threshing wheat grains are used as the control samples. The mold colonies and storage quality traits of wheat grains harvested by different threshing patterns are tested during storage.

## 2. Materials and Methods

### 2.1. Materials and Instruments

Two wheat cultivars, Huaimai 33 and Huaimai 35 were used as experiment samples, Huaimai 33 was hard red wheat cultivar, but Huaimai 35 was semi-hard white wheat cultivar. Wheat grains samples were harvested in the field plot of Jiangsu Reclamation Agriculture Development Co., Ltd. Yellow Sea Branch, Yancheng City, Jiangsu Province, China, in early June 2019. Wheat grains of each cultivar were harvested in three patterns: manual threshing, axial flow threshing (axial flow threshing device (crop material flows along the axial line of the threshing drum), 4LBZ-172B PRO888GM Kubota, Kubota Agricultural Machinery (Suzhou) Co., Ltd., Suzhou, China) and tangential flow threshing (tangential flow threshing device (crop material flows along the tangential line of the threshing drum), C230 John Deere (Jiamusi), Agricultural Machinery Co., Ltd., Jiamusi, China). Each wheat grains sample was a mixture of 3 different sampling points, and each sample weighed 10 kg; 6 samples were obtained. The broken wheat grains and impurity were removed. All samples were dried to safe storage moisture content (13.0%) under shade condition and stored in refrigerator at temperature 4 °C for six months. The moisture content of wheat grains samples was adjusted to 18% before storage experiment, then wheat grains samples were stored in an artificial climate box at 30 °C and 80% RH for 40 days [2].

The instruments used in this study mainly included the following: upright pressure steam sterilizer (model, LS-35HD, Jiangyin Binjiang Medical Equipment Co., Ltd., Wuxi, China) used to sterilize, thermostatic water bath box (model, HH-2, Bonsai Instrument Technology Co., Ltd. Shanghai, China) used to retain potato dextrose Agar in liquid, artificial climate box (model, RGQ-360N, Shanghai Senxin Experimental Instrument Co., Ltd., Shanghai, China) used to cultivate mold colonies, slap type aseptic homogenizer (model, ZGJZQ-10, Jintan Jincheng Shuohua instrument Factory, Changzhou, China) used to separate the mold colonies from grains sample, upright cleaning table (model, SP-DJ, Shanghai Pudong physical and chemical instrument factory, Shanghai, China), electronic balance (model, UTP-313, accuracy, 0.01 g, Shanghai Huachao Electric Appliance Co., Ltd., Shanghai, China), automatic counting particle machine (model, SLY-G, Zhejiang Top Instrument Co., Ltd., Hangzhou, China) used to count grains and a laboratory pure water machine (model, RO-10, Nanjing Qianyan Instrument Equipment Co., Ltd., Nanjing, China).

The reagents used in this study mainly included the following: PDA (potato dextrose Agar) (Shanghai Ruiqi Industrial Co., Ltd., Shanghai China), sterile diluent (0.85% sterile saline), TTC (Triphenyltetrazolium Chloride) (Beijing Tianhong Shengshi Trading Co., Ltd., Beijing, China), benzene (Shanghai Jiuyi Chemical Reagent Co., Ltd., Shanghai, China), 95% ethanol (Shanghai Jiuyi Chemical Reagent Co., Ltd., Shanghai, China), 0.01 mol/L Potassium hydroxide standard titrant (Shanghai Jiuyi Chemical Reagent Co., Ltd., Shanghai, China) and 0.04% phenolphthalein ethanol solution (Tianjin Chemical Reagent Research Institute Co., Ltd., Tianjin, China).

### 2.2. Research Methods

#### 2.2.1. Experiment for Counting Mold Colonies of Wheat Grains

The plates, test tubes, suction head and PDA were sterilized by using pressure steam sterilizer at 121 °C for 15 min before the experiment. PDA was put into the water bath box at temperature 46 °C after sterilization. According to GB 4789.15-2016 [29], counting mold colonies of wheat grains was carried out on the vertical cleaning table. The stored 25 g grains sample was taken into an aseptic homogeneous bag and 225 mL sterile diluent was added into the aseptic homogeneous bag, then, the wheat sample was homogenized in tapping type sterile homogenizer (homogenization frequency 12 times/s, homogenization time 3 min). The homogenized wheat sample solution was diluted in an incremental series of 10-folds; two or three diluted wheat sample solutions were selected. 1 mL solution from each diluted wheat sample solution was taken and placed, respectively, in two plates, and 20–25 mL PDA was poured into each plate. The plate was turned to mix evenly until the PDA solidified, then the plate was placed in the artificial climate box under 28 °C for 5 days, and the mold colonies were counted according to the colony morphology.

The tests were repeated three times; the results were averaged. The mold colonies of wheat were counted at 10 days interval during storage.

#### 2.2.2. Experiment for Testing Wheat Seed Vigor

After 100 wheat grains were soaked in warm water for 18 h, the seed embryos were cut longitudinally by a scalpel. Then seed embryos were soaked in TTC dye and were put in artificial climate box at 30 °C for dark time 14 h. Seed embryos were washed repeatedly with ultra-pure water after soaking, stained seed embryos were observed by using a magnifying glass, the stained red seed embryos were counted [30]. The tests were repeated three times. Seed vigor was detected at 10 days interval during storage.

#### 2.2.3. Experiment for Testing Dry Basis 1000 Grain Weight

1000 wheat grains were obtained by counting particle machine, the wheat grains were dried in drying box at 130 °C for 24 h. The wheat grains weight was measured with an electronic balance after drying, this was the dry basis 1000 grain weight according to GB/T 5519-2018 [31] The tests were repeated three times. The dry basis 1000 grain weight was detected at 10 days interval during storage.

#### 2.2.4. Experiment for Testing Germination Characteristics

Experiments for testing germination characteristics of wheat grains were carried out before and after storage according to GB/T3543.4-1995 [32]. The germination potential was recorded on the 4th day, the germination rate, the aberrations and the mold colonies on seedling root were recorded on the 8th day.

#### 2.2.5. Experiment for Testing Fatty Acid Value

Fatty acid value of wheat grains was tested according to GB/T5510-2011 [33] during storage. The tests were repeated three times. The fatty acid value was tested at 10 days interval during storage.

### 2.3. Statistical Analysis of Data

SPSS 24.0 software was used for significant test and correlation analysis. The significant test used the least significant difference (LSD) test for multiple comparisons, significant test set the significant level at 0.05, correlation analysis set the correlation level at 0.01.

## 3. Results

### 3.1. Characteristics of Mold Colonies of Wheat Grains

**Figure 1:**
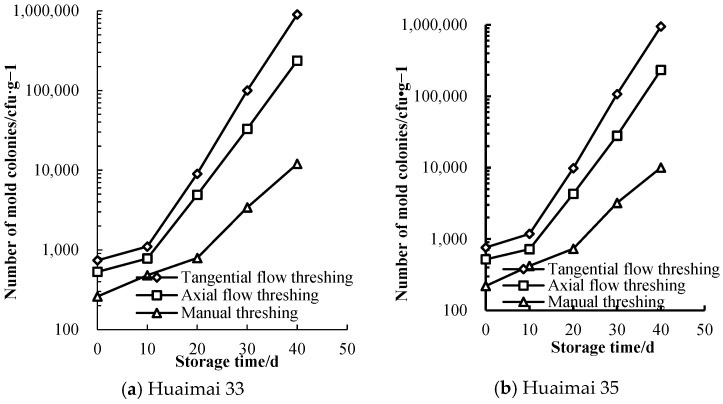
Mold colonies of wheat grains harvested by different threshing patterns during storage.

The mold colonies of wheat grains harvested by different threshing patterns were counted during the storage process, the results were shown in Figure 1. There was a significant difference in mold colonies of wheat grains harvested by different threshing patterns before storage (*p* < 0.05), the mold colonies of wheat grains harvested by TFT was greater than that by AFT, and that by AFT was greater than that by manual threshing. The mold colonies of wheat grains harvested by different threshing patterns was less than 10^3^ cfu·g^−1^, the mold colonies didn’t reach the mildewed standard of wheat grains (less than 10^3^ cfu·g^−1^) and the wheat grains was in safe storage [2].

During the storage process, the mold colonies of wheat grains increased with increasing storage time. The mold colonies of wheat grains harvested by TFT were greater than that by AFT, and that by AFT was greater than that by manual threshing. The increased mold colonies of wheat harvested by TFT was greater than that by AFT, and that by AFT was greater than that by manual threshing. The mold colonies of wheat grains harvested by TFT also increased faster than that by AFT.

Wheat grains harvested by TFT reached the slightly mildewed standard on the 10th day, closed to the mildly mildewed standard on the 20th day, reached the severely mildewed standard on the 30th day and exceeded the severely mildewed standard on the 40th day. The wheat grains harvested by AFT did not reach the slightly mildewed standard on the 10th day, reached the slightly mildewed standard on the 20th day, closed to the mildly mildewed standard on the 30th day and reached the severely mildewed standard on the 40th day. The wheat grains harvested by manual threshing did not reach the slightly mildewed standard on the first 20th day, reached the slightly mildewed standard on the 30th day and close to the mildly mildewed standard on the 40th day.

After storage, there was a significant difference in mold colonies of wheat grains harvested by different threshing patterns (*p* < 0.05), the mold colonies of wheat grains harvested by TFT was greater than that by AFT and that by AFT was greater than that by manual threshing. The mold colonies of wheat grains harvested by TFT increased by 1250 times; mold colonies of wheat grains harvested by AFT increased by 448 times during the storage. The mold colonies of Huaimai 33 and Huaimai 35 wheat grains harvested by different threshing patterns were approximate before and after storage.

### 3.2. Characteristics of Wheat Seed Vigor

**Figure 2:**
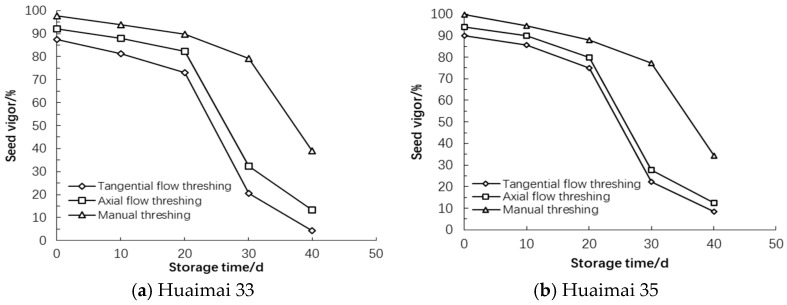
Seed vigor of wheat harvested by different threshing patterns during storage.

The seed vigor of wheat grains harvested by different threshing patterns was detected during the storage (Figure 2), there was a significant difference in seed vigor of wheat grains harvested by different threshing patterns before storage (*p* < 0.05). Seed vigor of wheat grains harvested by manual threshing was greater than that by AFT and that by AFT was greater than that by TFT. The difference in seed vigor of wheat grains was related to mechanical damage. Threshing force acted on wheat grains during threshing by machine and resulted in the mechanical damage to wheat grains; and seed vigor reduced. The seed vigor of Huaimai 35 wheat grains harvested by different threshing patterns was higher than that of Huaimai 33 after harvesting.

The seed vigor of wheat grains harvested by different threshing patterns decreased with increasing storage time. The seed vigor of wheat grains harvested by TFT decreased faster than that by AFT. Decreasing extent of seed vigor of wheat grains harvested by manual threshing was less than that by AFT, and that by AFT was less than that by TFT during the storage.

After storage, there was a significant difference in seed vigor of wheat grains harvested by different threshing patterns (*p* < 0.05). The seed vigor of wheat grains harvested by TFT reduced 83.00%, that by AFT reduced 81.67%. The seed vigor of Huaimai 35 wheat grains harvested by different threshing patterns reduced more than that of Huaimai 33 after storage except TFTP.

Compared with manual threshing, mechanical threshing significantly reduced seed vigor of wheat grains (*p* < 0.05). The seed vigor of wheat grains harvested by TFT was significantly lower than that by AFT (*p* < 0.05) after storage. Mechanical damage on wheat seed embryo caused wheat seed vigor to be reduced before storage, the mildewed wheat significantly reduced catalase activity during the storage and seed lost vigor further [34].

### 3.3. Characteristics of Wheat Dry Basis 1000 Grain Weight

There was no significant difference in dry basis 1000 grain weight of wheat between TFT and AFT before storage (*p* > 0.05), but there was a significant difference in dry basis 1000 grain weight of wheat between mechanical threshing and manual threshing before storage (*p* < 0.05). The dry basis 1000 grain weight loss of Huaimai 35 wheat grains harvested by different threshing patterns was higher than that of Huaimai 33 after harvesting.

**Figure 3:**
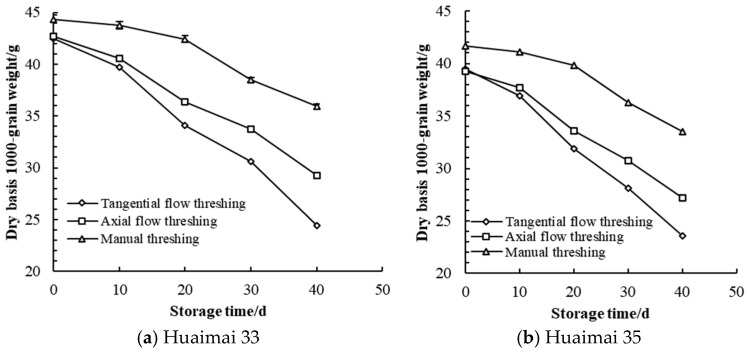
Dry basis 1000 grain weight of wheat harvested by different threshing patterns during storage.

The dry basis 1000 grain weight of wheat grains harvested by different threshing patterns was tested during storage (Figure 3). The dry basis 1000 grain weight of wheat grains harvested by manual threshing during storage was greater than that by AFT, and that by AFT was greater than that by TFT. The dry basis 1000 grain weight of wheat grains decreased with increasing storage time, the decreasing extent of dry basis 1000 grain weight of wheat harvested by TFT was greater than that by AFT, and that by AFT was greater than that by manual threshing. The dry basis 1000 grain weight of wheat grains harvested by TFT decreased faster than that by AFT.

After storage, there was a significant difference in the dry basis 1000 grain weight among wheat grains harvested by different threshing patterns (*p* < 0.05), the dry basis 1000 grain weight of wheat grains harvested by manual threshing was greater than that by AFT, the dry basis 1000 grain weight of wheat grains harvested by AFT was greater than that by TFT. The dry basis 1000 grain weight of wheat grains harvested by TFT reduced 18.08 g, the dry basis 1000 grain weight of wheat grains harvested by AFT reduced 13.38 g. During the storage, mold activity needed to hydrolyze carbohydrates of wheat grains, hydrolyzing carbohydrates meant that the starch content of wheat grains decreased [5], non-reducing sugar decreased and reducing sugar increased. Reducing sugar was used as the substrate of mold respiration, so the dry basis 1000 grain weight of wheat grains decreased [35]. The dry basis 1000 grain weight loss of Huaimai 33 wheat grains harvested by different threshing patterns was larger than that of Huaimai 35 after storage.

### 3.4. Germination Characteristics

The Germination characteristics of wheat grains harvested by different threshing patterns before and after storage were shown in Table 1. The seed quality of wheat grains harvested by mechanical threshing patterns was lower than that by manual threshing pattern before storage. It meant that mechanical damage to wheat grains affected the wheat seed quality. The seed quality of wheat grains harvested by three threshing patterns all reduced after storage.

There were significant differences in the aberrations and the mold colonies in seedling root of wheat grains harvested by different threshing patterns before and after storage (*p* < 0.05). The aberrations and the mold colonies in seedling root of wheat grains by TFT was greater than that by AFT, and that by AFT was greater than that by manual threshing. Compared with manual threshing, the aberrations and the mold colonies in seedling root of wheat grains harvested by TFT increased 15.67% and 20.00%, respectively, before storage, and that increased 31.00% and 23.66%, respectively, after storage.

There were significant differences in the germination potential and germination rate of wheat grains harvested by different threshing patterns before and after storage (*p* < 0.05), the germination potential and germination rate of wheat grains harvested by manual threshing were larger than those by AFT and those by AFT were larger than those by TFT. Compared with manual threshing, the germination potential and germination rate of wheat grains harvested by TFT decreased 16.34% and 16.34%, respectively, before storage, and that decreased 30.67% and 31.00%, respectively, after storage. The Germination characteristics of Huaimai 33 and Huaimai 35 wheat grains harvested by different threshing patterns were approximate before and after storage.

The germination potential and germination rate of wheat grains harvested by TFT reduced 76.67% and 77.67%, respectively, after storage, the aberrations and the mold colonies in seedling root of wheat grains harvested by TFT increased 79.00% and 66.66%, respectively, after storage. The germination potential and germination rate of wheat grains harvested by AFT reduced 77.33% and 77.33%, respectively, after storage, the aberrations and the mold colonies in seedling root of wheat grains harvested by AFT increased 77.00% and 67.00%, respectively, after storage. The germination potential and germination rate of wheat grains harvested by manual threshing reduced 68.34% and 67.34%, respectively, after storage, the aberrations and the mold colonies in seedling root of wheat grains harvested by manual threshing increased 67.67% and 62.34%, respectively, after storage. The germination characteristics indexes of wheat grains harvested by TFT were lower than those by AFT after storage.

There were significant differences in the germination potential, germination rate, aberrations and mold colonies in seedling root of wheat grains harvested by different threshing patterns before storage, because the different threshing patterns caused the different threshing damage to wheat grains. There was no acting force on the wheat grains and no threshing damage to the wheat grains during manual threshing. The seed epidermis was intact, the mold infected and difficultly invaded the wheat grains interior during the storage.

### 3.5. Characteristics of the Fatty Acid Value in Wheat Grains

The fatty acid values of wheat grains harvested by different threshing patterns during storage were shown in Table 2. Fatty acid value was an indicator of the grain freshness, the fatty acid value of wheat grains increased gradually with the increasing storage time and the quality of wheat grains deteriorated with increasing storage time [36]. The fatty acid value of wheat grains harvested by mechanical threshing first increased and then decreased, while that by manual threshing increased consistently. At the same storage time, the fatty acid value of wheat grains harvested by TFT was higher than that by AFT; this meant that the quality of wheat grains harvested by TFT deteriorated faster than that by AFT during storage, the freshness of wheat grains harvested by TFTP was lower than that by AFTP during the storage.

During the storage, the fatty acid value of wheat grains increased with increasing storage time under high temperature and high humidity conditions [37] when wheat grains seriously mildewed, microbes decomposed fatty acid and the fatty acid value decreased [38]. The fatty acid value of wheat grains was related to the threshing patterns of wheat grains during storage. The fatty acid values of Huaimai 33 and Huaimai 35 wheat grains harvested by different threshing patterns were approximate before and after storage.

### 3.6. Analysis of Correlation Between Wheat Storage Quality Traits and Mold Colonies of Wheat Grains

#### 3.6.1. Analysis of Correlation Between Seed Vigor and Mold Colonies of Wheat Grains

**Figure 4:**
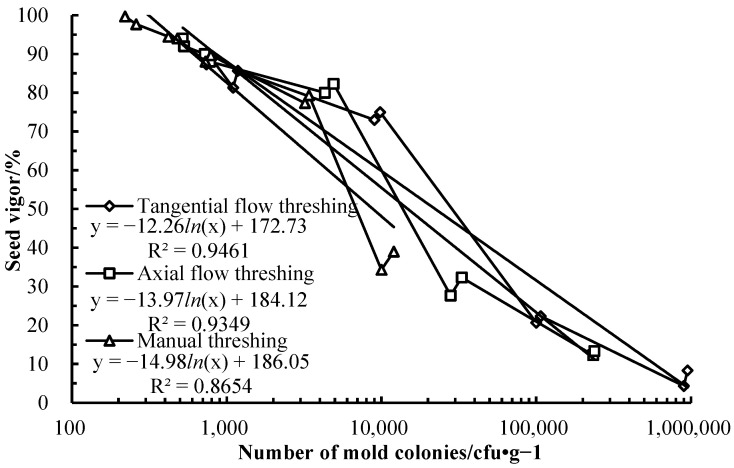
Correlation between seed vigor and mold colonies of wheat grains during storage.

During the storage process, the correlation between seed vigor and mold colonies of wheat grains was shown in Figure 4. The logarithmic negative correlation equation between the mold colonies and seed vigor of wheat grains harvested by TFT was y = 172.76 − 12.26 *In*(x), determination coefficient *R*^2^ was 0.9461 (*p* < 0.001), the logarithmic negative correlation equation between the mold colonies and seed vigor of wheat grains harvested by AFT was y = 184.04 − 13.96 *In*(x), determination coefficient *R*^2^ was 0.9349 (*p* < 0.001), the logarithmic negative correlation equation between the mold colonies and seed vigor of wheat grains harvested by manual threshing was y = 185.59 − 14.95 *In*(x), determination coefficient *R*^2^ was 0.8654 (*p* < 0.001). The mold colonies greatly affected seed vigor. The seed vigor of wheat grains reduced logarithmically with the mold colonies of wheat grains increasing.

#### 3.6.2. Analysis of Correlation Between the Dry Basis 1000 Grain Weight and Mold Colonies of Wheat Grains

**Figure 5:**
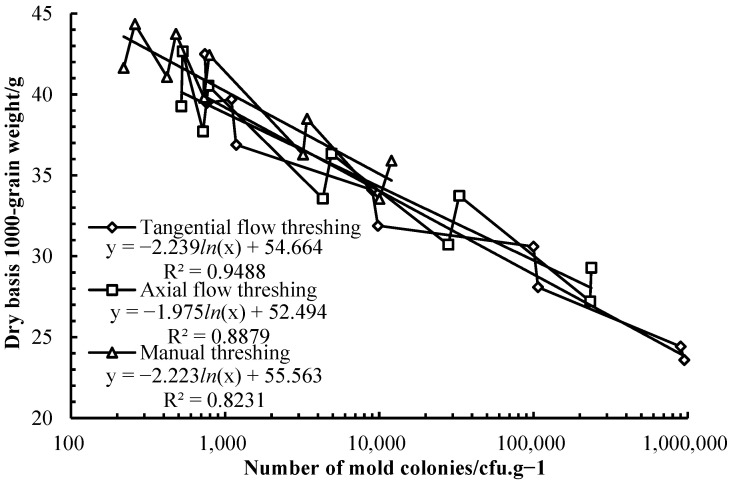
Correlation between dry basis 1000 grain weight and mold colonies of wheat grains during storage.

During the storage process, correlation between the dry basis 1000 grain weight and mold colonies of wheat grains was shown in Figure 5. The logarithmic negative correlation equation between the mold colonies and dry basis 1000 grain weight of wheat grains harvested by TFT was y = 54.676 − 2.24 *In*(x), determination coefficient *R*^2^ was 0.9488 (*p* < 0.001), the logarithmic negative correlation equation between the mold colonies and dry basis 1000 grain weight of wheat grains harvested by AFT was y = 52.118 − 1.933 *In*(x), determination coefficient *R*^2^ was 0.8879 (*p* < 0.001), the logarithmic negative correlation equation between the mold colonies and dry basis 1000 grain weight of wheat grains harvested by manual threshing was y = 55.482 − 2.212 *In*(x), determination coefficient *R*^2^ was 0.8231 (*p* < 0.001). The mold colonies affected greatly the dry basis 1000 grain weight of wheat grains. The mold activity needed to consume substance, hydrolase was produced when the molds multiplied and starch was hydrolyzed for the mold activity. The starch content of wheat grains decreased [34] and the dry basis 1000 grain weight of wheat grains reduced [35] or dry matter losses increased [39]. The dry basis 1000 grain weight of wheat grains reduced with the mold colonies of wheat grains increasing.

#### 3.6.3. Correlation Analysis Between Germination Characteristics Indexes and Mold Colonies of Wheat Grains

The correlation analysis between germination characteristics indexes and mold colonies of wheat grains was shown in Table 3 before and after storage.

Before storage, the germination potential and germination rate were significantly negative correlation with the mold colonies of wheat grains, correlation coefficients were 0.964, 0.965, respectively, the aberrations and the mold colonies in seedling root were significantly positive correlation with the mold colonies of wheat grains, correlation coefficients were 0.962, 0.962, respectively. After storage, the germination potential and germination rate were still significantly negative correlation with the mold colonies of wheat grains, correlation coefficients were 0.825, 0.829, respectively, the aberrations and the mold colonies in seedling root still had a significantly positive correlation with the mold colonies of wheat grains, correlation coefficients were 0.826, 0.904, respectively. By comparing the correlation coefficients, it was concluded that there was a significant correlation between the mold colonies and germination characteristics indexes of wheat grains before and after storage.

## 4. Discussion

Threshing is the most important process at harvesting wheat grains and threshing damage to wheat grains is related to the type [17,24], the structural and operating parameters of threshing device [17]. The speed of AFT drum is 505 r·min^−1^ and the length of AFT drum is 1000 mm. The speed of TFT drum with axial flow plate separation drum structure is 800~900 r·min^−1^, the length of threshing and separation drum is 3344 mm. Acting force of TFT drum on wheat grains is larger than that of AFT drum, and the acting time of TFT drum on wheat grains is longer than that of AFT drum. Studies show that mechanical damage of AFT drum to rice grains is less than that of TFT drum [21], the mold colonies of rice harvested by TFT drum are more than that by AFT drum [22]. The mold colonies of wheat grains harvested by TFT drum increase faster than that by AFT drum and the mold colonies of wheat grains harvested by TFT drum are more than that by AFT drum during the storage. Mold infects wheat grains damaged by the threshing machine more easily and grows faster on damaged wheat grains during storage.

This study analyzes the storage quality of wheat grains damaged by different threshing patterns under artificial storage conditions. There are significant correlations between mold colonies and storage quality of wheat grains. There exist mold colonies on wheat grains surface in spite of harvesting patterns or threshing patterns [6]. Although mold colonies distribute on the surface of wheat grains [9], once wheat grains or surface of wheat grains were damaged, mold colonies more easily invaded into the interior of the wheat grains. Mold colonies deteriorate the quality of storage wheat [5]. This study also manifests a fact that the mold colonies number and dry basis 1000 grain weight loss of wheat grains harvested by threshing machine are higher than those by man. Avoiding and reducing damage to wheat grains will be a particularly important consideration during the threshing process. The damaged wheat grains should not be stored for long time and should be stored under strictly controlled conditions.

The study substantiates that the wheat grains damaged by threshing machine seriously mildew and quality of damaged wheat grains seriously deteriorates during storage process. Only parts of quality traits, dry basis 1000 grain weight, seed vigor, germination characteristics index and the fatty acid value of wheat grains, are tested in this study, more quality traits, fungal diversity, distribution of microbial contamination within wheat grains, et al., should be tested in future research [9,10]. The action mechanism of fungal on the wheat epidermis also deserves further investigation.

Research has manifested that soft wheat has better storage tolerance than harder cultivars [40], but no obvious difference on the wheat storage quality between the hard and semi-hard wheat grains harvested by different threshing patterns is found in this study. Mechanical damage properties and storage quality traits of hard and soft wheat cultivars harvested by different threshing patterns should be researched further in future.

## 5. Conclusions

(1) The mold colonies, dry basis 1000 grain weight, seed vigor, germination characteristics index and fatty acid value of wheat grains by AFT had significant difference with TFT before and after storage. The mold colonies, the fatty acid value, the aberrations and the mold colonies in seedling root of wheat grains harvested by TFTP increased faster than those by AFTP, the dry basis 1000 grain weight, the seed vigor, the germination potential and germination rate of wheat grains harvested by TFTP decreased faster than those by AFTP.

(2) There was a significant correlation between mold colonies and germination characteristics index of wheat grains (*α* < 0.01). The greater the mold colonies of wheat grains, the lower the seed vigor of wheat grains, and these were the significant logarithmic correlations between the mold colonies and seed vigor of wheat grains harvested by TFT and between the mold colonies and seed vigor of wheat grains harvested by AFT. The greater the mold colonies of wheat grains, the lower the dry basis 1000 grain weight of wheat grains, and these were the significant logarithmic correlations between the mold colonies and dry basis 1000 grain weight of wheat grains harvested by TFT, and between the mold colonies and dry basis 1000 grain weight of wheat grains harvested by AFT. The AFTP should be preferred for mechanical harvesting wheat grains in terms of the storage safety and storage quality of wheat grains.

(3) There were certain differences between hard wheat and semi-hard wheat on the seed vigor and the dry basis 1000 grain weight loss before and after storage. There were no certain differences between hard wheat and semi-hard wheat on the mold colonies number, the germination characteristics and the fatty acid values before and after storage.

## Figures and Tables

**Table 1:** Germinating characteristics indexes of wheat grains harvested by different threshing patterns (%).

Cultivar	Threshing Methods	Germination Potential	Germination Rate	Aberrations	Mold Colonies in Seedling Root
Huaimai 33	TFT (before storage)	81.00 ± 2.65 ^c^	81.67 ± 2.08 ^c^	16.67 ± 1.53 ^a^	32.67 ± 4.04 ^a^
	TFT (after storage)	3.33 ± 0.58 ^c^	4.00 ± 0.00 ^c^	95.67 ± 0.58 ^a^	99.33 ± 0.58 ^a^
	AFT (before storage)	86.67 ± 2.31 ^b^	87.33 ± 3.06 ^b^	11.00 ± 4.00 ^b^	26.33 ± 1.58 ^b^
	AFT (after storage)	12.00 ± 0.00 ^b^	13.67 ± 0.58 ^b^	86.67 ± 0.58 ^b^	89.67 ± 0.58 ^b^
	Manual threshing (before storage)	97.00 ± 2.00 ^a^	97.67 ± 1.53 ^a^	1.00 ± 1.00 ^c^	14.00 ± 2.00 ^c^
	Manual threshing (after storage)	34.00 ± 0.00 ^a^	35.00 ± 1.00 ^a^	64.67 ± 0.58 ^c^	75.67 ± 0.58 ^c^
Huaimai 35	TFT (before storage)	82.33 ± 1.53 ^c^	82.33 ± 1.53 ^c^	17.00 ± 1.00 ^a^	32.33 ± 2.89 ^a^
	TFT (after storage)	7.33 ± 1.53 ^c^	8.00 ± 1.73 ^c^	91.67 ± 1.53 ^a^	98.33 ± 0.58 ^a^
	AFT (before storage)	89.00 ± 1.00 ^b^	89.33 ± 1.15 ^b^	10.33 ± 1.53 ^b^	23.33 ± 3.06 ^b^
	AFT (after storage)	11.67 ± 0.58 ^b^	12.00 ± 0.00 ^b^	87.33 ± 0.58 ^b^	90.33 ± 0.58 ^b^
	Manual threshing (before storage)	98.67 ± 0.58 ^a^	98.67 ± 0.58 ^a^	1.33 ± 0.58 ^c^	12.33 ± 0.58 ^c^
	Manual threshing (after storage)	30.33 ± 0.58 ^a^	31.33 ± 1.16 ^a^	69.00 ± 1.00 ^c^	74.67 ± 0.58 ^c^

Note: Values within a column followed by different small letters are significantly different at 5% probability levels among different treatments of a wheat cultivar.

**Table 2:** Detection of fatty acid value of wheat grains harvested by different threshing patterns during storage (mg·(100 g)^−1^).

Cultivar	Storage Time (d)	TFT	AFT	Manual Threshing
Huaimai 33	0	19.2	19.2	17.9
	10	25.9	23.2	19.3
	20	55.0	50.9	37.8
	30	91.4	88.4	51.3
	40	86.9	85.4	85.2
Huaimai 35	0	15.4	15.4	14.1
	10	22.0	20.6	19.3
	20	48.5	44.4	36.5
	30	96.7	92.5	42.2
	40	88.3	82.7	89.2

**Table 3:** Correlation analysis between mold colonies and germination characteristics indexes of wheat grains harvested by different threshing patterns before and after storage (*p* < 0.01).

Wheat Germination Characteristics Indexes Before Storage	Correlation Coefficient	Wheat Germination Characteristics Indexes After Storage	Correlation Coefficient
Germination potential	−0.964 **	Germination potential	−0.825 **
Germination rate	−0.965 **	Germination rate	−0.829 **
Aberrations	0.962 **	Aberrations	0.826 **
Mold colonies in seedling root	0.962 **	Mold colonies in seedling root	0.904 **

Note: ** significant at *α* < 0.01.

## Data Availability

The original contributions presented in the study are included in the article, further inquiries can be directed to the corresponding author.

## References

[1] Shiju M. (2010). A review on the effect of fungi on the wheat grain under post-harvest storage ecology. Food Environ. Saf.—J. Fac. Food Eng..

[2] Zhao Y.J., Han X.X., Guo W., Zhang J., Zheng X.L., Tian J.Z. (2013). Study on influence of mildewing on wheat quality. J. Henan Univ. Technol. (Nat. Sci. Ed.).

[3] Wang R.L., Kong X.G. (2011). Study on the changes and relativity of husked rice physiological activities under controlled atmosphere storage conditions. J. Henan Univ. Technol. (Nat. Sci. Ed.).

[4] Sun H., Jiang W.L., Nei L. (2011). Changes of physiological activity and quality of wheat during storage. Cereals Oils Food Sci. Technol..

[5] Schmidt M., Horstmann S., De Colli L., Danaher M., Speer K., Zannini E., Arendt E.K. (2016). Impact of fungal contamination of wheat on grain quality criteria. J. Cereal Sci..

[6] Qi Z., Zhou X., Tian L., Zhang H., Lei Y., Tang F. (2024). Assessment of wheat grain quality in China during harvest and storage using a mycobiome approach. Food Secur..

[7] Xu H.Y. (2013). The Effect of Temperature Fluctuation on Stored-Wheat Safe Moisture Content and Microbial Activity.

[8] Wei X. (2012). The Studies on Activity Characteristics of Fungus on Different Varieties of Wheat in the Process of Storage.

[9] Adriana L., Zoe M., Mario D., Colin W., Severino S.P. (2006). Distribution of microbial contamination within cereal grains. J. Food Eng..

[10] Sabina M.T., Renata H.B., Patrícia Z., Fábio d.S.G., Benedito C. (2016). Fungal diversity and natural occurrence of deoxynivalenol and zearalenone in freshly harvested wheat grains from Brazil. Food Chem..

[11] Senatore M.T., Prodi A., Tini F., Balmas V., Infantino A., Onofri A., Cappelletti E., Oufensou S., Sulyok M., Covarelli L. (2023). Different diagnostic approaches for the characterization of the fungal community and Fusarium species complex composition of Italian durum wheat grain and correlation with secondary metabolite accumulation. J. Sci. Food Agric..

[12] Zhang Y.Y., Pei F., Fang Y., Li P., Xia J., Sun L., Zou Y.Y., Shen F., Hu Q.H. (2019). Interactions among Fungal Community, Fusarium Mycotoxins, and Components of Harvested Wheat under Simulated Storage Conditions. J. Agric. Food Chem..

[13] Wang R.H., Li M.M., Jin R., Liu Y.X., Guan E.Q., Mohamed S.R., Bian K. (2024). Interactions among the composition changes in fungal communities and the main mycotoxins in simulated stored wheat grains. J. Sci. Food Agric..

[14] Qi Z.H., Tian L., Zhang H.Y., Zhou X., Lei Y.Q., Tang F. (2024). Mycobiome mediates the interaction between environmental factors and mycotoxin contamination in wheat grains. Sci. Total Environ..

[15] Wang R.L., Liu L.L., Guo Y.P., He X., Lu Q. (2020). Effects of deterioration and mildewing on the quality of wheat seeds with different moisture contents during storage. RSC Adv..

[16] Chen Z., Wassgren C., Kingsly Ambrose R.P. (2020). A review of grain kernel damage: Mechanisms, modeling, and testing procedures. Trans. ASABE.

[17] Derevjanko D., Holovach I., Bulgakov V., Kuvachev V., Olt J. (2020). Theoretical and experimental research into impact of threshing tools in combine grain harvesters on quality of cereal crop seeds. Agron. Res..

[18] Xu L.Z., Li Y.M., Ma Z., Zhao Z., Wang C.H. (2013). Theoretical analysis and finite element simulation of a rice kernel obliquely impacted by a threshing tooth. Biosyst. Eng..

[19] Lachuga Y.F., Bur’yanov A.I., Pakhomov V.I., Chervyakov I.V. (2020). Adaptation of threshing devices to physical and mechanical characteristics of harvested crops. Russ. Agric. Sci..

[20] Gu R.L., Huang R., Jia G.Y., Yuan Z.P., Ren L.S., Li L., Wang J.H. (2019). Effect of mechanical threshing on damage and vigor of maize seed threshed at different moisture contents. J. Integr. Agric..

[21] Li Y.N., Chen J.S., Ding Q.S., Ding W.M. (2017). Effects of axial flow and tangential flow mechanical threshing on rice damage and processing quality. Trans. Chin. Soc. Agric. Eng..

[22] Li L., Li Y.N., Chen Y.L., Ding Q.S., He R.Y. (2020). Study on storage quality of japonica rice by different mechanical threshing and its relevance. Sci. Technol. Food Ind..

[23] Neme K., Mohammed A. (2017). Mycotoxin occurrence in grains and the role of postharvest management as a mitigation strategies. A review. Food Control.

[24] Masek J., Kumhala F., Novak P., Fic T. Influence of different threshing system design on grain damage. Proceedings of the 15th International Scientific Conference Engineering for Rural Development.

[25] Wei L.J. (2016). The Design of Plot Wheat Breeding Threshing Machine and Research on It’s Low Damage Characteristics.

[26] Javad K., Feizollah S., Jafar M., Mehdi N., Mohammad H.K. (2008). Evaluation and modeling of physical and physiological damage to wheat seeds under successive impact loadings: Mathematical and neural networks modeling. Crop Sci..

[27] Som B., Pao S., late Hourn S., Yim S., Sareth C., Makara O., Chao S., Nob L., Hel S., Yong R. (2019). Fissured grain and head rice yield of crops harvested manually or by combine at different ripening stages in Cambodia. Plant Prod. Sci..

[28] Warzecha T., Zieliński A., Skrzypek E., Wójtowicz T., Moś M. (2012). Effect of mechanical damage on vigor, physiological parameters, and susceptibility of oat (*Avena sativa*) to *Fusarium culmorum* infection. Phytoparasitica.

[29] (2016). National Food Safety Standard-Microbiological Examination of Food-Count of Molds and Yeasts.

[30] Bi X.H., Dai X.W. (2011). Seed Science.

[31] (2018). Grains and Pulses-Determination of the Mass of 1000 Grains.

[32] (1995). Rules for Agricultural Seed Testing-Germination Test.

[33] (2011). Inspection of Grain and Oils-Determination of Fat Acidity Value of Grain and Oilseeds.

[34] Wan Z.M., Wu L. (2005). The wheat quality deterioration in different storage temperature. Cereals Oils Food Sci. Technol..

[35] Xu Z.F., Zhang H.Y., Zhang D.Y. (2000). Wheat Quality and Its Improvement.

[36] Ma H., Zhang L.J., Liu C. (2013). Fatty acid value as an index of wheat storage quality control. Food Process..

[37] Tian P.P., Lv Y.Y., Yuan W.J., Zhang S.B., Hu Y.S. (2019). Effect of artificial aging on wheat quality deterioration during storage. J. Stored Prod. Res..

[38] Guo L.L. (2006). The change law on fatty acid value of stored wheat flour. Grain Storage.

[39] Garcia-Cela E., Kiaitsi E., Sulyok M., Medina A., Magan N. (2018). *Fusarium graminearum* in stored wheat: Use of CO_2_ production to quantify dry matter losses and relate this to relative risks of zearalenone contamination under interacting environmental conditions. Toxins.

[40] Zhang S.B., Lv Y.Y., Wang Y.L., Jia F., Wang J.S., Hu Y.S. (2017). Physiochemical changes in wheat of different hardnesses during storage. J. Stored Prod. Res..

